# Lactate-Fortified *Puerariae Radix* Fermented by *Bifidobacterium breve* Improved Diet-Induced Metabolic Dysregulation via Alteration of Gut Microbial Communities

**DOI:** 10.3390/nu12020276

**Published:** 2020-01-21

**Authors:** Yura Choi, Shambhunath Bose, Na Rae Shin, Eun-Ji Song, Young-Do Nam, Hojun Kim

**Affiliations:** 1 Department of Rehabilitation Medicine of Korean Medicine, Dongguk University, 27 Donggukro, Ilsan-donggu, Goyang 10326, Korea; youla21@naver.com (Y.C.); sskr1207@nate.com (N.R.S.); 2 BexPharm Healthcare Ltd., Seoul 04781, Korea; shambose@yahoo.com; 3 Research Group of Gut Microbiome, Korea Food Research Institute, Wanju-gun 24 55365, Korea; songej486@hanmail.net (E.-J.S.); youngdo98@kfri.re.kr (Y.-D.N.); 4 Department of Food Biotechnology, Korea University of Science and Technology, Wanju-gun 34113, Korea

**Keywords:** *Pueraria lobata*, anti-inflammatory, metabolic syndrome, anti-obesity, beta-diversity, alpha-diversity

## Abstract

Background: *Puerariae Radix* (PR), the dried root of *Pueraria lobata*, is reported to possess therapeutic efficacies against various diseases including obesity, diabetes, and hypertension. Fermentation-driven bioactivation of herbal medicines can result in improved therapeutic potencies and efficacies. Methods: C57BL/6J mice were fed a high-fat diet and fructose in water with PR (400 mg/kg) or PR fermented by *Bifidobacterium breve* (400 mg/kg) for 10 weeks. Histological staining, qPCR, Western blot, and 16s rRNA sequencing were used to determine the protective effects of PR and fermented PR (fPR) against metabolic dysfunction. Results: Treatment with both PR and fPR for 10 weeks resulted in a reduction in body weight gain with a more significant reduction in the latter group. Lactate, important for energy metabolism and homeostasis, was increased during fermentation. Both PR and fPR caused significant down-regulation of the intestinal expression of the MCP-1, IL-6, and TNF-α genes. However, for the IL-6 and TNF-α gene expressions, the inhibitory effect of fPR was more pronounced (*p* < 0.01) than that of PR (*p* < 0.05). Oral glucose tolerance test results showed that both PR and fPR treatments improved glucose homeostasis. In addition, there was a significant reduction in the expression of hepatic gene PPARγ, a key regulator of lipid and glucose metabolism, following fPR but not PR treatment. Activation of hepatic AMPK phosphorylation was significantly enhanced by both PR and fPR treatment. In addition, both PR and fPR reduced adipocyte size in highly significant manners (*p* < 0.001). Treatment by fPR but not PR significantly reduced the expression of PPARγ and low-density lipoproteins in adipose tissue. Conclusion: Treatment with fPR appears to be more potent than that of PR in improving the pathways related to glucose and lipid metabolism in high-fat diet (HFD)+fructose-fed animals. The results revealed that the process of fermentation of PR enhanced lactate and facilitated the enrichment of certain microbial communities that contribute to anti-obesity and anti-inflammatory activities.

## 1. Introduction

Metabolic dysregulation, which represents a constellation of metabolic abnormalities, (e.g., hyperglycemia, hyperinsulinemia, hyperlipidemia, etc.), is a vital indicator of obesity-related diseases such as insulin resistance, type 2 diabetes, and fatty liver disease [1]. More specifically, in addition to systemic inflammation, metabolic dysregulation is a key complication of obesity [2]. The major contributing factor to obesity is thought to be an imbalance between energy intake and energy expenditure. More explicitly, obesity is associated with over-eating as well as with the consumption of nutrient-poor foods containing saturated fats and high levels of sugar accompanied by a reduced level of physical activity [3].

An accumulation of evidence indicates the possible therapeutic implication of natural agents such as probiotics, prebiotics, and phytochemicals to improve obesity and metabolic dysfunction [4,5,6,7]. *Puerariae Radix* (PR), the dried root of *Pueraria lobata*, is widely used in traditional Chinese medicine and is also consumed as food. This medicinal herb, which has recently been included in Western dietary supplements, is known to possess protective effects against various diseases including metabolic diseases such as obesity, diabetes, and hyperlipidemia. Previous studies have reported that chronic administration of PR extract can decrease serum total cholesterol and blood pressure [8], fasting plasma glucose levels [9], and lipid levels in liver and serum [10], as well as improving glucose tolerance [9] and insulin signaling [8], and increasing adenosine triphosphate (ATP) levels and glucose uptake [11].

Fermentation is a microorganism-mediated process that breaks down or converts undesirable substrates into compatible components, thereby improving substrate properties via the production and enrichment of bioactive compounds [7]. A growing line of evidence indicates the beneficial health effects of probiotics and their fermented food products [12]. Fermentation-driven bioactivation of herbal medicines can result in improved therapeutic potencies and efficacies and decreased toxicities in locations where the microbial population has a pivotal role [7]. Recently, there has been rapid progress in microbial fermentation techniques as a result of in-depth research on the modernization of herbal medicines. Accordingly, microbial-mediated fermentation and transformation of herbal drugs have drawn considerable attention as new approaches to the production of novel active compounds with potent medicinal values [13]. Indeed, it has been shown that the process of fermentation can enhance the anti-inflammatory activities of herbs through a number of mechanisms [7,14,15]. These include, but are not limited to, the suppression of NF-κB activation and inhibition of the translocation of active NF-κB p65 through reduced IκBα degradation as well as the phosphorylation of extracellular signal-regulated kinase (ERK) and p38 and c-Jun NH2-terminal kinase (JNK) mitogen-activated protein kinase (MAPK) [9]. In some cases, such improvement in the anti-inflammatory activity was observed to be NF-κB-independent and demonstrated to be mediated via the mammalian target of rapamycin (mTOR)/p70 S6 kinase pathway and other unknown pathways [9]. Moreover, an accumulation of evidence indicates that augmentation of the activities of herbs against inflammation through fermentation is mediated via modulation of gut microbial communities [14,16,17]. Additionally, several previous studies have indicated the beneficial effects of fermentation on the anti-obesity properties of herbal medicines [15,17]. In a previous study, soymilk fermented by *Bifidobacterium breve* was demonstrated to improve alcohol metabolism, lipid metabolism, and reduce mammary carcinogenesis in mouse models [18]. Furthermore, *Bifidobacterium* sp. and its rice-fermented beverage were observed to efficiently suppress adipogenesis and lipogenesis, promote lipid catabolism, improve glucose-insulin homeostasis, and prevent obesity [19].

Gut microbes appear to have an important role in human metabolism and health [20]. They also take part in the maintenance of balance of the host’s metabolism and immune modulation systems [21]. An imbalance in the composition of the gut microbiota (dysbiosis) has been associated with various clinical conditions including obesity [22]. The probable mechanisms by which the gut microbiota could cause the onset and development of obesity and the related metabolic diseases include: (a) high abundance of carbohydrate-fermenting bacteria which leads to an increased production of short-chain fatty acids (SCFAs), providing an extra source of energy for the host in the form of stored lipids or glucose; (b) augmented intestinal permeability to bacterial lipopolysaccharides (LPS), resulting in elevated systemic LPS levels that trigger low-grade inflammation and insulin resistance; and (c) enhanced activity of the gut endocannabinoid system [23]. A number of our previous studies have shown that the protective effects of several herbal formulations against inflammatory insult and metabolic disorders are mediated via modulation of the gut microbial population [17,24,25,26,27,28,29].

Although PR is known to produce a number of beneficial effects, the detailed mechanism(s) of the actions involved has not been properly understood. Additionally, fermented PR (fPR) was shown to be more effective than unfermented PR in ovariectomized rats [30]. However, the effects of PR and fPR against metabolic dysregulation have not been reported in detail. In the present study, we evaluated the effectiveness of protection by PR and fPR against metabolic dysfunction in a high-fat diet (HFD) plus fructose (HFD+fructose)-induced female mouse model. The rationale behind the selection of female mice for this evaluation is based on a previous study in which female subjects exhibited more body fat than their male counterparts [31]. Moreover, menopause, a biological factor that affects fat distribution, may augment the risk or exacerbate the negative effects of obesity on health in females [32]. Additionally, the effects of PR and fPR on the distribution profile of mouse intestinal microbiota were studied in order to elucidate the possible mechanism(s) by which these two herbal formulations may exert their beneficial effects.

## 2. Materials and Methods

### 2.1. Herbal Preparation

Dried root of *P. lobata* in powdered form was purchased from the medical supply store of Dongguk University International Hospital (Ilsan, Goyang-si, Gyeonggi-do, Korea). To prepare the PR extract, 500 g of PR powder was mixed vigorously with 5 L of 30% ethanol (*v*/*v*), and the resultant mixture sonicated in a water bath for 1 h at room temperature. The mixture was then centrifuged at 1200× *g* for 15 min at room temperature following which the supernatant was filtered through a Whatman^®^ Grade 4 filter paper (Whatman, Maidstone, Kent, UK). The residue was subjected to the above-mentioned extraction procedure twice using 40 mL of 30% ethanol (*v*/*v*) each time. The collected extract was evaporated to dryness using a rotary evaporator (EYELA N-1200A, EYELA, Tokyo, Japan) and subsequently freeze-dried using a lyophilizer (Bondiro, IlshinBioBase, Dongducheon-si, Gyeonggi-do, Korea). The resultant product was kept at −80 °C until further use.

The *B. breve* used in this study as a starter for PR fermentation was obtained from Cellbiotech (Gimpo, Gyeonggi-do, Korea). Selected pure *B. breve* was incubated in MRS broth media (BD Difco ^™^, Franklin Lakes, NJ, USA) at 37 °C for 24 h. The broth was then centrifuged at 12,000× *g* at room temperature for 3 min and the supernatant discarded. The cell pellet was washed with PBS (pH 7.4) three times. Finally, the bacterial cells were resuspended in PBS and inoculated to a PR extract prepared in distilled water for 24 h.

### 2.2. High-Performance Liquid Chromatography (HPLC)-Based Analysis of PR and fPR

Chromatographic analysis of PR and fPR was carried out using an HPLC system (1260 infinity, Agilent Technologies, Santa Clara, CA, USA) equipped with a UV detector, a column oven, and an autosampler. Sulfuric acid at a concentration of 0.008 N was used as the mobile phase. Separation of the samples was achieved on an Eclipse XDB-C18 column (5 µm, 250 mm × 4.6 mm, Agilent Technologies) at 25 °C with the mobile phase flow rate maintained at 0.6 mL/min. Detection of the ingredients was carried out at 210 nm using malic acid, lactic acid, and citrate acid (Sigma-Aldrich, Saint Louis, MO, USA) as standards for calibration (Figure S1).

### 2.3. Animals and Treatment

The animal study was approved by the Institutional Animal Care and Use Committee of Dongguk University and performed in accordance with the Guide for the Care and Use of Laboratory Animals (Institute of Laboratory Animal Resources, Commission on Life Sciences, National Research Council, USA; National Academy Press: Washington D.C., 1996). Forty female C57BL/6J mice (4-weeks-old, bodyweight 18 ± 1 g) were purchased from Daehan Biolink Co. Ltd. (Eumseong, Chungbuk, Republic of Korea). The animals were acclimatized for 1 week under a 12 h light/dark cycle at constant temperature (25 °C) and humidity (50–60%) levels and were provided with free access to standard chow diet (Feedlab, Guri-si, Gueonggi-do, Korea) and water. The mice were then allocated into five groups of eight animals each as follows: normal, HFD+F, Orlistat (XEN), PR, and fPR groups. The mice in the normal group were provided with a normal diet (Research Diets Inc., New Brunswick, NJ, USA) while the animals in the other four groups were fed 60% HFD feed (Research Diets Inc.) and 20% fructose (F) in water for 12 weeks. From Week 2 to Week 12 of this feeding schedule, the mice in the XEN group were treated orally with the anti-obesity drug Orlistat (Xenical^®^, Roche, Milano, Italy; 10 mg/kg/day) as a positive control. This drug blocks the digestion of dietary triglycerides by inhibiting gastric and pancreatic lipases and is known to promote weight loss and improve obesity-related risk factors in obese subjects with and without metabolic complications [33]. The animals in the PR and fPR groups were exposed to PR (400 mg/kg/day) or fPR (400 mg/kg/day), respectively. This dose was selected based on previous reports [34,35]. The mice in the normal and HFD+F groups were orally administered with water as the vehicle. The treatment with the above-mentioned drugs and vehicle were performed five times per week. Following the termination of the treatment schedule at Week 12, mice of all groups were food-deprived overnight but provided free access to water. Generally, overnight fasting is applied in animal experiments in a typical metabolic study, probably because it is recommended in human medical examinations [36,37,38,39,40]. It has been reported in mice that overnight fasting nearly depletes liver glycogen stores. This event has the advantage of minimizing variability in the baseline blood glucose level [36,41]. Furthermore, an overnight fast of 18 h in mice has led to increased insulin sensitivity when compared to that from a 5 h fast [41]. The mice were then sacrificed under anesthesia induced by the intraperitoneal administration of Zoletil^®^ (Tiletamine-zolazepam, Virbac, Carros, France) and Rompun^®^ (xylazine-hydrochloride, Bayer, Leverkusen, Germany), each at a dose of 1 mL/kg. Blood was immediately collected from the animals in a BD Vacutainer^®^ SST™ tube (BD, Franklin Lakes, NJ, USA) and allowed to clot for 30 min at room temperature. Serum was separated by centrifuging the blood samples at 3000 r/min for 15 min and finally stored at −80 °C until use. Liver, intestine, and adipose tissues were excised quickly, washed in ice-cold PBS (pH 7.4), blotted, and weighed. Some portions of the tissues were immediately kept either alone (for immunoblotting) or in Invitrogen™ RNAlater™ stabilization solution (Thermo Fisher Scientific, Waltham, MA, USA, for RNA preparation) and then snap-frozen in liquid nitrogen before storing at −80 °C until further use. Other portions of the tissues, required for histological analysis, were fixed immediately in 4% formalin (Junsei, Tokyo, Japan) and stored at 4 °C until being further processed.

### 2.4. Quantitative Real-Time PCR (qRT-PCR)

Total RNA was extracted from the stored tissues using a commercial TRIzol^®^ reagent kit (Life Technologies, Carlsbad, CA, USA) and following the kit manufacturer’s instructions. The qualitative and quantitative analyses of the extracted RNA were carried out by obtaining optical measurements at 260 nm and 280 nm using a nanodrop spectrophotometer (Implen, Munich, Germany). The cDNA was synthesized by reverse transcription of equal quantities of each RNA sample (1 µg) using an oligo-(dT) 18 cDNA RT PreMix kit (Bioneer, Daejeon, Korea).

The qRT-PCR was performed on a Light Cycler 480^TM^ platform (Roche Applied Science, Basel, Switzerland) in a 96-well plate using SYBR Green master mix (Toyobo, Tokyo, Japan). The amplification reactions were carried out following the kit manufacturer’s instructions in a total 20 µL volume of PCR mixture, containing 1 µL of cDNA, 10 pmol of each reverse and forward primer of the selected gene (Bioneer; Table S1), 10 µL of SYBR Green master mix, and 8 µL of nuclease-free water. The following conditions were applied for PCR amplification: an initial denaturation step at 95 °C for 10 min followed by 45 cycles of amplification involving denaturation at 95 °C for 10 s, annealing at 55–58 °C for 5 s, and extension at 72 °C for 10 s. After completion of this reaction, melting curve analysis was carried out to determine the purity and specificity of the amplicon. All amplification reactions were performed in duplicate, and the data were processed and analyzed using dedicated Light Cycler software (version 1.2, Roche Applied Science) and normalized using glyceraldehyde-3-phosphatase dehydrogenase (GAPDH) as the housekeeping gene. The relative gene expression levels were quantified following the standard 2^−∆*C*t^ estimation method, in which *C*_t_ represents the crossing threshold value derived by the software with ∆*C*_t_ = (*C*_t-target gene_ − *C*_t-GAPDH_).

### 2.5. Immunoblotting

Liver tissues were homogenized on ice in RIPA buffer containing protease inhibitor (Sigma-Aldrich) and phosphatase inhibitor cocktail (GenDEPOT, Barker, TX, USA) by using a Vibra-Cell™ ultrasonic liquid processor (Sonics & Materials, Newtown, CT, USA). The tissue homogenates were centrifuged at 14,000 r/min for 30 min at 4 °C to eliminate the insoluble materials. The resultant supernatants were collected and stored at −80 °C until use. The protein concentrations of the supernatants were determined by using a bicinchoninic acid (BCA) protein assay kit (Thermo Scientific, Rockford, IL, USA) per the instructions of the kit manufacturer. Thirty micrograms of protein were subjected to denaturation at 100 °C in Laemmli sample buffer (BioRad, Hercules, CA, USA) containing 5% β-mercaptoethanol. The protein was then resolved by SDS-PAGE using a constant voltage of 100 V for 90 min and finally transferred to a 0.45 μm polyvinylidene fluoride (PVDF) membrane (Amersham, Buckinghamshire, UK) using a Mini Trans-Blot^®^ electrophoretic transfer cell device (BioRad, Hercules, CA, USA). The membranes were incubated with Tris-buffered saline (Sigma-Aldrich) containing 0.1% Tween 20 (TBST) and 5% non-fat dried milk (Becton Dickinson, Sparks, MD, USA) for 30 min. Following this, the membranes were washed three times with TBST and finally incubated overnight with anti-phospho-AMPK (Thr 172) antibody (Cell Signaling Technology, Danvers, MA, USA) and anti-phospho-AKT (S473) antibody (Cell Signaling Technology, Danvers, MA, USA) at 4 °C in TBST containing 5% non-fat dried milk. The membranes were washed twice with TBST and then incubated for 90 min with the appropriate horseradish peroxidase-conjugated anti-IgG secondary antibody (Cell Signaling Technology) (1:2000 dilution in TBST containing 1% non-fat dried milk) The immunoreactive bands were detected by using a BioRad ChemiDoc XRS imaging system (BioRad) with a Super Signal West Pico ECL reagent (Thermo Fisher Scientific, San Jose, CA, USA). Band densities were analyzed using ImageJ software (National Institutes of Health, Bethesda, MA, USA). Subsequently, the membranes were stripped in a buffer (62.5 mM Tris-HCl [pH 6.7] containing 2% SDS and 100 mM β-mercaptoethanol), re-probed with anti-AMPK antibody (Cell Signaling Technology) and anti-AKT antibody (Cell Signaling Technology), and processed identically as described above. All membranes were finally re-probed in a similar way with β-actin as the housekeeping protein and using an anti-β-actin antibody (Cell Signaling Technology).

### 2.6. H&E Staining

The formalin-fixed liver tissues were dehydrated in a series of increasing ethanol concentrations and then embedded in paraffin blocks. The tissues were cut into 6 μm thick sections using a microtome (Leica RM2235, Leica, Nussloch, Germany). The obtained sections were placed on silicon-coated glass slides (Leica Biosystem, Richmond, IL, USA) and dried. The sections were then mounted on slides and stained with hematoxylin (Sigma-Aldrich) and eosin Y (Sigma-Aldrich) as previously described [39]. The stained tissue sections were examined under an Olympus BX61 microscope (Olympus, Tokyo, Japan), and the images were captured at 200× magnification using an Olympus DP70 cooled digital color camera (Olympus).

### 2.7. Oral Glucose Tolerance Test (OGTT)

The OGTT was performed 48 h prior to the termination of the experimental schedule. For this test, 18 h-fasting-adapted mice were administered a sterilized glucose solution (Sigma-Aldrich) by oral gavage at a dose of 2 g/kg body weight. Blood was collected from the tail vein of the animals at five different post-treatment times (0, 30, 60, 90, 120 min). Serum glucose concentrations were measured at five different post-treatment times (0, 30, 60, 90, 120 min) using an ACCU-CHECK Active system (ACCU-CHEK, Mannheim, Germany). The OGTT results are expressed in area under the curve (AUC) terms in order to determine the degree of impairment of glucose tolerance.

### 2.8. Sequencing of 16S rRNA Gene Amplicon of Fecal Microbiota

Bacterial DNA from stool samples was isolated using a QIAamp stool DNA mini kit (QIAGEN, Hilden, Germany) by applying the method described in our previous report [39]. PCR of the V1-V3 region of the 16s rRNA gene sequences was carried out using a C1000 Touch thermal cycler with a 96-deep-well reaction module (Biorad, Hercules, CA, USA). The PCR products were purified using a LaboPass PCR purification kit (COSMO GENTECH, Seoul, Korea). The amplicons of each sample were pooled in equimolar amounts and then purified using AMPure XP beads (Agencourt Bioscience, Beverly, MA, USA) and finally quantified using a PicoGreen dsDNA assay kit (Invitrogen, Carlsbad, CA, USA). The mixed amplicons were amplified on sequencing beads by undertaking emulsion PCR. The sequencing reactions were conducted on a Roche/454 GS Junior system (454 Life Sciences, Branford, CT, USA) following the manufacturer’s instructions.

### 2.9. Sequence Analysis

Initially, the sequence reads were filtered to reduce the number of errors; low-quality reads (average quality score <20 or read length <300 bp) were eliminated from further analysis. The sequences were then processed and the operational taxonomic units (OTUs) were clustered using the open reference OTU picking method (at 97% sequence similarity) in accordance with the Quantitative Insights into Microbial Ecology (QIIME) pipeline (version 1.9.1) [42]. Alpha-diversity was calculated using the observed OTUs and the Chao1 estimator. The total structural changes in the gut microbial communities were analyzed using UniFrac-based principal coordinated analysis (PCoA) to reveal the clustering pattern of the microbial composition in each experimental group. To profile the taxa with differing abundances among the groups, a linear discriminant analysis effect size (LEfSe) assessment was performed using an online program [43]. For that purpose, the threshold of the logarithmic linear discriminant analysis (LDA) score was set to >2.0, and the alpha value of the factorial Kruskal–Wallis test among classes was set to <0.05.

### 2.10. Statistical Analysis

All experimental data are expressed as mean ± SEM values unless otherwise indicated. Statistical significance was evaluated by applying the *t*-test or one-way analysis of variance (ANOVA) followed by Tukey’s post-hoc test using GraphPad Prism 5. The strength of a relationship between parameters was assessed using the two-tailed Pearson’s correlation test. A correlation was considered significant only when the absolute value of Pearson’s correlation coefficient *r* was greater than 0.5.

## 3. Results and Discussion

### 3.1. Analysis of fPR Preparation

**Figure 1:**
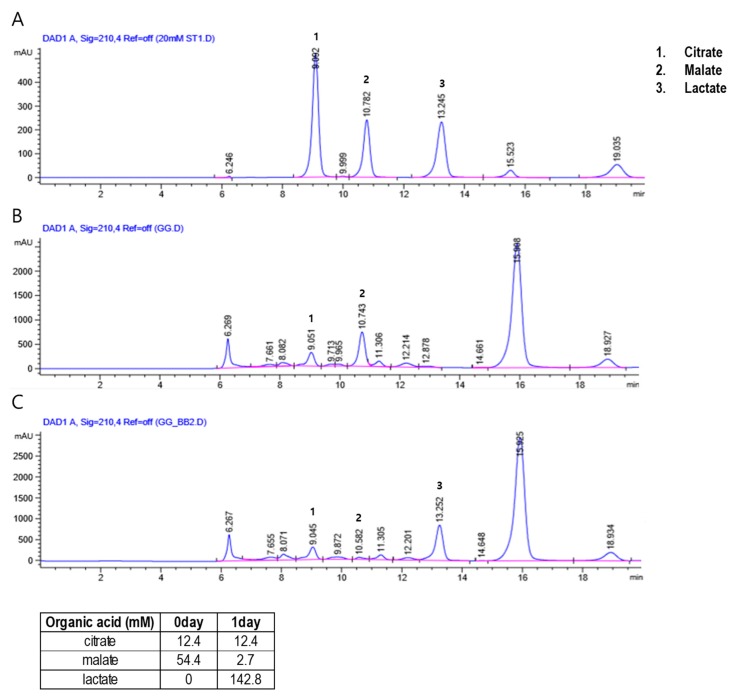
High-performance liquid chromatography (HPLC) chromatograms showing the content of (1) citrate, (2) malate, and (3) lactate in the standard solution used for calibration (**A**), and the levels of *Puerariae Radix* (**B**) and fermented *Puerariae Radix* (**C**) in the herbal preparations.

The HPLC-based organic acid analysis showed that the concentration of malate decreased from 54.4 mM to 2.7 mM while that of lactate increased from 0 mM to 142.8 mM in the PR extract after subjecting it to *B. breve-*mediated fermentation (Figure 1). This suggests a possible bacterial conversion of the malate from PR to lactate, which can be catalyzed by a number of enzymes including malate dehydrogenase (MDH) and malolactic enzyme (MLE). The presence of these two enzymes in *Bifidobacterium* species has been reported in earlier studies [44,45]. Lactate has been suggested to function as an active metabolite and to have important roles in muscle glycogen production and muscle fatigue, as well as in regulatory activities such as the modulation of energy production [46] and energy homeostasis [47].

### 3.2. Effects of PR and fPR on the Body Weight, Liver Weight, and Intestinal and Total Fat Weights in the HFD+F Group

**Figure 2:**
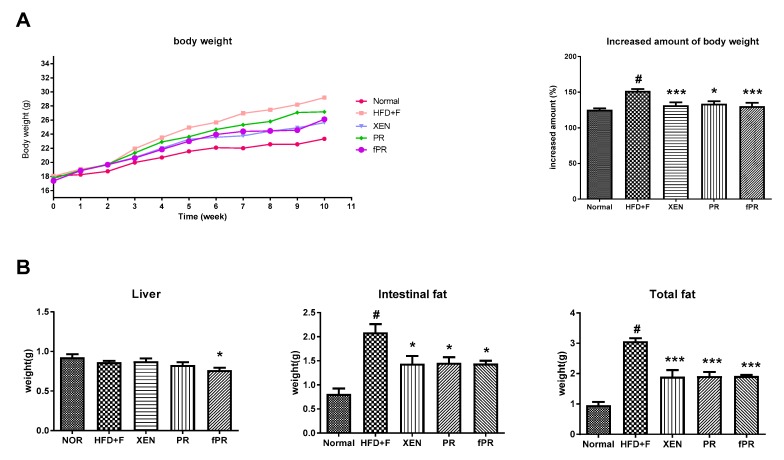
Effects of *Puerariae Radix* (PR) and fermented *Puerariae Radix* (fPR) on body weight gain, liver weight, and intestinal and total fat weights of normal and high-fat diet plus fructose (HFD+F)-fed mice during the 10 week study period: (**A**) body weight and (**B**) tissue weights as indicated. Bodyweight was recorded every week. Tissue weights were recorded at sacrifice. Data are expressed as means ± SD; differences were evaluated by using one-way ANOVA. # *p* < 0.05 compared to the normal group; * *p* < 0.05, *** *p* < 0.001 compared to the HFD+F group.

At the termination of the study (10th-week post-treatment), no significant change in food intake was evident among the experimental groups (data not shown). However, at this time, body weight gain and the intestinal and total body fat weights were significantly higher in the HFD+F-fed animals than in the normal group (*p* < 0.05) (Figure 2A,B). However, liver weight remained unchanged among the groups (Figure 2B). In rodents, a HFD is reported to induce obesity resembling that in the human metabolic syndrome, which is characterized by increased body weight along with induction of steatosis, impaired glucose and lipid metabolism, and low-grade inflammation [48]. Interestingly, treatment of the HFD+F group with XEN, PR, or fPR resulted in significant reductions in body weight gain, as well as intestinal fat and total fat weights (Figure 2B). The above results support those in a recent study that showed that treatment with a PR extract significantly reduced body and epididymal fat weights in HFD+F-treated mice [49]. Notably, in the current study, the body weight gain of the HFD+F-fed mice decreased more significantly in response to the fPR treatment (*p* < 0.001) than to the PR treatment (*p* < 0.05) (Figure 2A). It has previously been suggested that a fermented product of PR might be therapeutically more effective than unfermented PR and, therefore, may be useful as a functional food [30].

### 3.3. Anti-Inflammatory Effects of PR and fPR on the Liver, Intestine, and Intestinal Fat Tissue in HFD+F-Fed Animals

**Figure 3:**
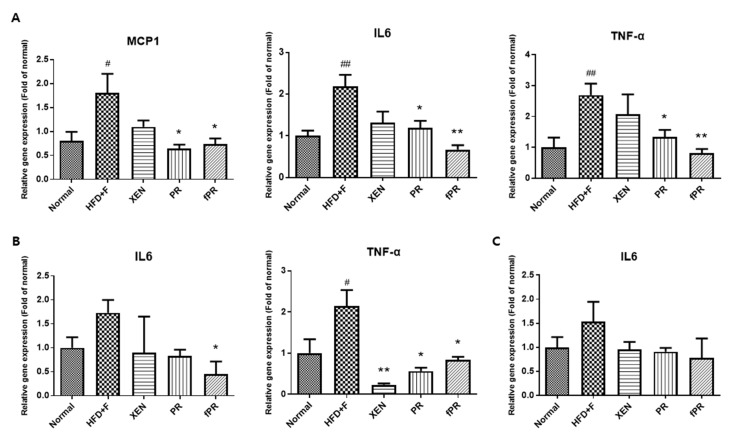
Expression of chemokine (MCP1) and pro-inflammatory cytokine (IL6 and TNFα) genes in (**A**) intestinal tissue, (**B**) intestinal fat tissue, and (**C**) liver tissue. Data are expressed as means ± SD, and differences were evaluated using one-way ANOVA. # *p* < 0.05, ## *p* < 0.01 compared to the normal group; * *p* < 0.05, ** *p* < 0.01 compared to the HFD+F-fed group.

Despite an overweight status and obesity being generally considered an outcome of an imbalance in energy homeostasis, several lines of evidence indicate that obesity is associated with alterations in immunity, such as chronic low-grade inflammation in which there are increased levels of circulating pro-inflammatory cytokines [50]. Crosstalk among the cellular constituents of adipose tissue, such as adipocytes, and endothelial and immune cells can result in an increase in inflammatory mediators such as TNF-α and IL-6. These ultimately exert important systemic effects, including negative regulation of normal insulin signaling via promotion of phosphorylation of insulin receptor substrates 1 (IRS-1) at serine residues [51,52,53]. In addition, it has been reported that a HFD increases the LPS level in serum, causing endotoxemia [54]. Moreover, there is evidence indicating that HFD can induce the production of inflammatory cytokines, including IL-1, IL-6, TNF-α, and MCP-1, in liver, muscle, and adipose tissues, leading to a low-grade inflammation that may be associated with obesity, insulin resistance, and other metabolic disorders [54]. In keeping with these findings, this study showed a significantly higher expression of the MCP-1 gene in the intestine of HFD+F-fed mice compared to that in the normal group (*p* < 0.05). A similar trend was evident in the expression of the TNF-α gene in the intestine and intestinal fat tissue. Moreover, compared to the normal group, IL6 gene expression was significantly higher (*p* < 0.01) in the intestine of the HFD+F group and was markedly higher in both the intestinal fat tissue and the liver of the HFD+F group (Figure 3A–C).

Notably, exposure of the HFD+F-fed mice to PR or fPR, but not XEN, resulted in significant down-regulation of the intestinal tissue expressions of the MCP-1, IL-6, and TNF-α genes (Figure 3A). Further, the inhibitory effect of fPR on the IL-6 and TNF-α gene expressions was more pronounced (*p* < 0.01) than that of PR (*p* < 0.05). In contrast, the intestinal fat tissue expression of the IL6 gene in the HFD+F-fed mice was significantly suppressed by fPR (*p* < 0.05), but not by XEN or PR. Additionally, co-treatment of the HFD+F-fed mice with XEN, PR, or fPR significantly down-regulated the intestinal fat tissue expression of the TNF-α gene and suppressed the expression of the IL6 gene in hepatic tissue, although the latter change was insignificant (Figure 3B,C).

Current lines of evidence indicate that the process of fermentation can potentiate the anti-inflammatory activities of herbs [55]. For example, we have shown that at a certain concentration, fermented *Rhizoma Atractylodis Macrocephalae* (RAM) could impose a significantly high level of inhibition on NO production compared to that from unfermented RAM in LPS-induced RAW 264.7 cells [27]. Furthermore, we have previously demonstrated a significant effect of fermentation on the improvement of an inhibitory effect of a *Rhizoma coptidis* extract on the LPS-induced expressions of iNOS, COX-2, TNF-α, IL-1β, and IL-6 genes in RAW 264.7 cells [14]. Additionally, compared to unfermented *Artemisia princeps* Pamp (AP), a fermented preparation of AP (FAP) more effectively reduced ear thickness and expressions of TNF-α, IL-1β, and IL-6 in a 12-*O*-tetradecanoylphorbol-13-acetate (TPA)-induced dermatitis mouse model [56].

### 3.4. Effects of PR and fPR on Adipocyte Size and Pathways Related to Glucose and Lipid Metabolism in the HFD+F Group

**Figure 4:**
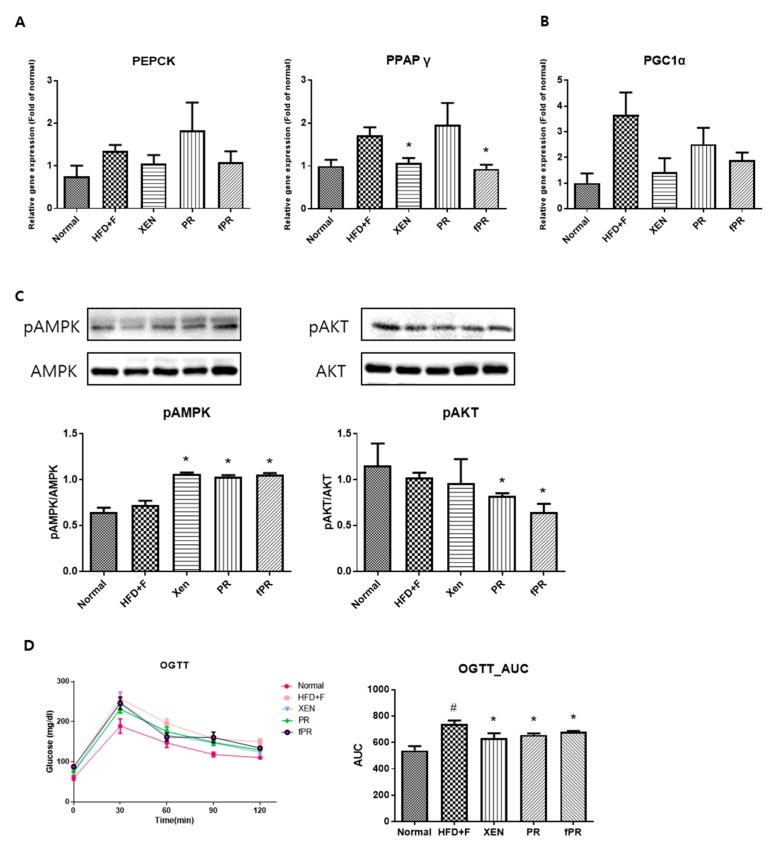
To confirm the effects of the herbal treatments on gluconeogenesis, gene expressions of key factors in the metabolic processes were assessed in hepatic and intestinal tissues. (**A**) Expressions of the PEPCK and PPARγ genes in the liver tissue, and (**B**) expression of the PGC1a gene in the intestinal tissue. (**C**) Phosphorylation of the AMPK and AKT proteins in the liver. (**D**) Results of the oral glucose tolerance test (OGTT) performed on the mice in the last week of the study. Areas under the curve (AUCs) were constructed as described in the ‘Materials and Methods’ section. Data are expressed as means ± SD and were evaluated using one-way ANOVA. # *p* < 0.05 compared to the normal group; * *p* < 0.05 compared to the HFD+F-fed group.

OGTT, a widely used procedure for evaluating whole-body glucose tolerance, has been employed in the assessment of insulin sensitivity [57]. Our OGTT results showed that fasting blood glucose levels were markedly higher at every measurement time (0, 30, 60, 90, and 120 min) in the HFD+F group than in the normal group. However, the glucose level in the HFD+F-fed mice improved when treated with XEN, PR, or fPR, as shown by the OGTT AUC results (Figure 4D). To explore the probable molecular mechanism(s) underlying such beneficial effects of XEN, PR, and fPR, we evaluated the effects of these agents in HFD+F-fed mice on the gene expressions associated with three different proteins PEPCK, PPARγ, and PGC1α, which have key roles in metabolic processes (Figure 4A,B). Among them, PEPCK, which catalyzes a committed, rate-limiting step in hepatic gluconeogenesis by converting oxaloacetate to phosphoenolpyruvate, has a vital role in maintaining a normal blood glucose level [58]. The nuclear receptor PPARγ is a regulator of lipid and glucose metabolism [59], whereas PGC1α, a multifunctional regulatory factor originally identified as a coactivator of PPARγ, has a central role in the regulatory network of glucose metabolism [60] and has been shown to be a metabolic regulator of intestinal epithelial cell fate [61]. In the present study, higher expressions of PEPCK and PPARγ genes in liver and a higher expression of PGC1α gene in intestine were observed in the HFD+F-fed mice compared to the expression levels in the normal group; however, the differences were statistically insignificant. Indeed, other studies have shown high hepatic gene and protein expressions of PEPCK and PPARγ in standard diet-fed mice compared to that in HFD-fed mice [62,63]. Moreover, PGC1α expression has been shown to be increased in the liver and pancreas of animal models with obesity and diabetes [64,65,66]. We noticed an insignificant but definite reduction in the hepatic expressions of PEPCK and PGC1α genes in the HFD+F-fed mice in response to treatment with XEN or fPR. An earlier study concluded that suppression of hepatic PEPCK expression may be a preventive or therapeutic strategy for metabolic diseases [67]. We also observed a significant reduction in the hepatic expression of PPARγ gene in the HFD+F group upon exposure to XEN and fPR, but not to PR. In an earlier study, using HepG2 cells as a model, we showed that down-regulation of PPARγ may contribute to the protective effect of herbal medicines against free fatty acids-induced hepatic steatosis [68]. On the other hand, AMPK, the function of which has been extensively studied in muscles and liver, has a central role in maintaining cellular energy homeostasis [8, 39, 69]. Activation of AMPK through phosphorylation induces pathways that increase energy production (glucose transport and fatty acid oxidation) and switches off pathways that utilize energy (lipogenesis, protein synthesis, and gluconeogenesis) [69]. Notably, in this study, phosphorylation of hepatic AMPK was significantly enhanced in the HFD+F-fed mice upon co-treatment with XEN, PR, or fPR (Figure 4C). This was accompanied by a significant reduction in the phosphorylation of AKT in both the PR and fPR groups compared to that in the HFD+F-fed mice (Figure 4C). Obesity has been reported to be a risk factor for nonalcoholic fatty liver disease (NAFLD), a disease spectrum characterized by excess fat accumulation in the hepatocytes leading to a number of pathophysiological conditions including hepatic steatosis (nonalcoholic fatty liver, NAFL), nonalcoholic steatohepatitis (NASH), and cirrhosis [70,71]. Excess hepatic accumulation of collagen via its synthesis (fibrogenesis) in the liver has a key role in the progression of NAFLD [72]. An accumulation of evidence indicates that PR possesses hepatoprotective activity and prevents liver fibrosis [73,74,75,76], and it has been reported that PR exerts an anti-fibrotic effect on the liver via suppression of the PI3K/AKT pathway to inhibit the over-accumulation of collagen [76].

**Figure 5:**
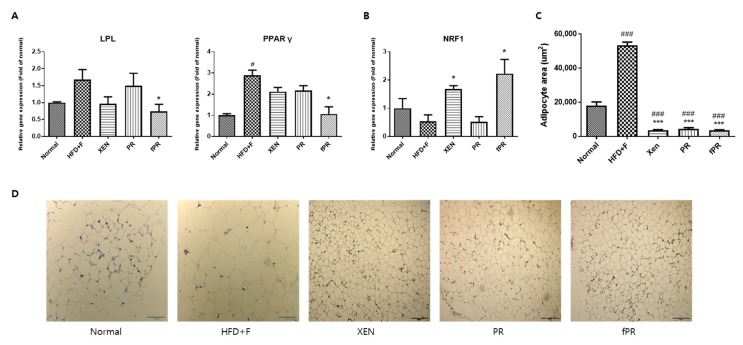
Effects of the herbal treatments on adipogenesis and gene expressions of key lipogenic factors and regulators in hepatic and intestinal fat tissues. (**A**) Expression of the LPL gene in liver tissue. (**B**) Expressions of the PPARγ and NRF1 genes in the intestinal fat tissue. (**C**,**D**) Intestinal fat tissue stained with hematoxylin and eosin; the sizes of the adipocytes are presented. Data are expressed as means ± SD and were evaluated using one-way ANOVA. # *p* < 0.05, ### *p* < 0.001 compared to the normal group; * *p* < 0.05, *** *p* < 0.001 compared to the HFD+F-fed group.

As expected, histological analysis of the intestinal adipose tissue showed that mice in the HFD+F group had significantly larger (*p* < 0.001) adipocytes than those in mice in the normal group (Figure 5C,D). In addition, there was significantly higher intestinal fat tissue expression of the PPARγ gene in the HFD+F group than in the normal group. These observations support those in a previous study on mice [77]. Moreover, hepatic and intestinal fat tissues showed higher and lower mRNA levels of LPL and NRF1, respectively, in the HFD+F group than in the normal group, although the differences were statistically insignificant (Figure 5A,B). PPARγ is reported to be an essential factor for adipogenesis and to have a key role in the maintenance of the differentiated state of adipocytes [78]. In addition, it has been reported that xanthigen, a nutraceutical combination used in weight management, can attenuate HFD-induced obesity through down-regulation of PPARγ in adipose tissue [79]. Indeed, there is recent evidence that PPARγ repression has the beneficial effects of reducing body weight and improving insulin sensitivity, suggesting a potential clinical role of PPARγ antagonists in obesity and type 2 diabetes [79,80]. On the other hand, LPL, a rate-limiting enzyme, catalyzes the hydrolysis of the triglyceride (TG) core of circulating TG-rich lipoproteins such as chylomicrons, low-density lipoproteins, and very-low-density lipoproteins [81]. Several lines of evidence indicate that overexpression of LPL causes insulin resistance [82] and promotes obesity [83]. NRF-1, a transcription factor, has a key role in the regulation of mitochondrial biogenesis; moreover, it has been shown to attenuate obesity and has been linked to the expression of genes involved in lipid metabolism [84]. In our study, co-treatment of the HFD+F group with XEN, PR, or fPR reduced the adipocyte size compared with that of the HFD+F group in a highly significant manner (*p* < 0.001) (Figure 5C,D), whereas exposure of the HFD+F group to fPR, but not XEN or PR, significantly reduced the intestinal adipose tissue expressions of both the PPARγ and LPL genes. On the other hand, the intestinal fat tissue mRNA level of NRF1 was significantly increased in the HFD+F-fed mice upon treatment with XEN or fPR, but not PR (Figure 5B). Taken together, these findings indicate it is conceivable that both PR and fPR can exert marked anti-obesity effects on a HFD+F-diet group. However, fPR appears to be more potent than PR in improving the pathways related to glucose and lipid metabolism in HFD+F-fed animals.

### 3.5. Effects of PR and fPR on the Gut Microbial Communities of HFD-Fed Mice

**Figure 6:**
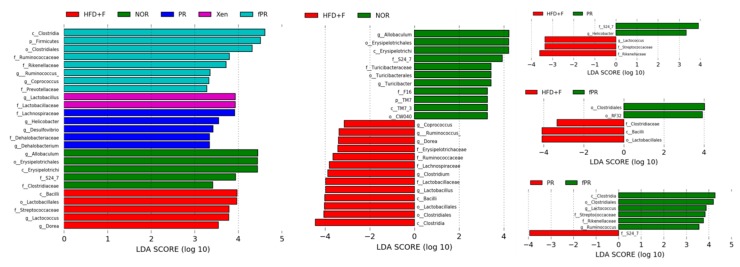
Linear discriminant analysis effect size (LEfSe) assessment of the bacterial communities in the mouse stool samples. The diversity of the gut microbiota was altered by feeding a HFD and fructose-supplemented water. The LEfSe plot shows enriched bacteria in all phenotypic categories. The alpha value for the factorial Kruskal–Wallis test is <0.05, and the threshold on the logarithmic LDA score for a discriminative feature is >2.0.

The LEfSe analysis of our 16S rRNA sequencing data showed distinct differences in the distributional patterns of the gut microbial populations between the normal and HFD+F groups (Figure 6). These results were further supported by our PCoA-based assessment of beta-diversity, which revealed a clear distinction between the gut microbial communities in the HFD+F group and that in the normal group. Additionally, alpha-diversity analysis, a measure of species richness based on our assessment, demonstrated that the Chao 1 index was lower in the HFD+F group than in the normal group, suggesting that gut microbial population diversity declined in response to HFD+F feeding (Figure S2). Diet is one of the critical factors that determine the gut microbial composition [85]. Several lines of evidence indicate that feeding animals a HFD alters gut microbial diversity and that the modulation of a gut microbial population is associated with an increased intestinal permeability due to gut barrier disintegration, which eventually manifests as the development of metabolic endotoxemia, inflammation, and metabolic disorders [26,86,87,88,89]. Earlier reports have shown that the gut microbiome influences host metabolism, which is mediated through a number of mechanisms such as catabolism of dietary toxins/carcinogens, fermentation of indigestible nutrients, synthesis of micronutrients, and enhancement of the absorption of electrolytes and minerals [90]. Moreover, it has been suggested that the gut microbiota of obese individuals may be more capable of extracting energy from a given diet than the gut microbiota of lean individuals [91,92], implying that the gut microbiota can act as an additional contributing factor to the pathophysiology of obesity [93].

The LEfSe profile further demonstrated that the populations of *Bacilli, Lactobacillales, Lachnospiraceae,* *Erysipelotrichaceae*, *Streptococcaceae, Lactococcus*, and *Dorea* were enriched in the HFD+F group (Figure 6). Higher levels of *Erysipelotrichaceae* have been associated with metabolic disorders, and an approximately 2.5-fold decrease in the abundance of *Erysipelotrichacea*e taxa in a hamster model of hypercholesterolemia was observed in response to treatment with an extract purported to improve cholesterol homeostasis [94,95]. Additionally, our results revealed that *Erysipelotrichaceae* abundance was positively correlated with body, liver, and intestinal weights, as well as with intestinal and adipose tissue expression levels of TNF-α and IL-6 and the hepatic LPL level (Figure S3). In parallel, we observed that *Lachnospiraceae* was positively and significantly correlated with body, liver, and intestinal weights, as well as with intestinal IL-6 and hepatic LPL levels (Figure S3). It has been reported that members of the *Lachnospiraceae* family are associated with the presence of type 2 diabetes and obesity [96]. In addition, our study showed that *Dorea* abundance was positively and significantly correlated with body, liver, and intestinal weights, as well as with intestinal and adipose tissue levels of IL-6 and the hepatic LPL level (Figure S3). This is in agreement with a previous report demonstrating that *Dorea* presence is associated with metabolic disorders [95].

Our assessment of beta-diversity also showed clear separation of the gut microbial communities of the XEN and PR groups from those of both the normal and HFD+F groups. In contrast, the gut microbial distribution pattern in the fPR group was approximately similar to that in the HFD+F group (Figure S2B–D). In keeping with these results, the alpha-diversity, in terms of the Chao 1 index, in both the XEN and PR groups matched very closely to that in the normal group, whereas the fPR group exhibited an alpha-diversity pattern very similar to that in the HFD+F group. Additionally, the LEfSe profile showed that the abundance of the S24_7 family was higher in both the PR and normal groups than that in the HFD+F group. Moreover, a larger population of *Helicobacter* was observed in the PR group than in the HFD+F group (Figure 6). Abundances of both of these gut microbes were observed to be positively and significantly correlated with several of the obesity- and inflammatory-related parameters evaluated in this study (Figure S3). Previous studies have shown that S24_7 family abundance increases in mouse gut in response to a HFD [97]. In addition, *Helicobacter pylori* abundance has been associated with obesity in a clinical study [98]. These finding suggest that despite similarities in the alpha-diversity of gut microbial distribution profiles between the normal and PR groups, there was enrichment of certain microbial communities in the PR group that are related to obesity. A comparison of the LEfSe results between the PR and fPR groups demonstrated enrichment of *Lactococcus* and *Ruminococcus* in the latter group (Figure 6). Abundances of both of these genera were negatively and significantly correlated with most of the examined obesity- and inflammatory-related parameters (Figure S3). This suggests that despite the alpha-diversity of the gut microbial distribution pattern in the fPR group matching closely with that of the HFD+F group, the PR fermentation process facilitated the enrichment of certain microbial communities that contribute to anti-obesity and anti-inflammatory activities.

## 4. Conclusions

In summary, our results suggest that both PR and fPR have beneficial effects on HFD+F-induced metabolic dysregulation. However, fPR appears to have greater potency than PR for improving the functioning of pathways related to glucose and lipid metabolism in HFD+F-fed mice. Lactate was fortified during the PR fermentation process, which might facilitate the enrichment of certain microbial communities that contribute to anti-obesity and anti-inflammatory activities.

## Supplementary Materials

The following are available online at https://www.mdpi.com/2072-6643/12/2/276/s1, Figure S1: HPLC calibration curve of the organic acid standards; Figure S2: (A) Alpha-diversity, measured by determining the CHAO1 index. (B, C, D) PCoA score plots calculated from OTU levels by QIIME pipeline and subjected to unweighted UniFrac analysis; Figure S3. Hierarchical clustering presented as a heat-map shows the abundance of representative OTUs related to biomarkers (greatest difference between the gut microbiota and the biomarkers) selected for *p* < 0.05; Table S1: Nucleotide sequences of primers used in quantitative real-time PCR.

## Abbreviations

PR	*Puerariae Radix*
fPR	Fermented Puerariae Radix
HFD	High-fat diet
XEN	Orlistat
MCP1	Monocyte chemoattractant protein 1
IL-6	Interleukin 6
PEPCK	Phosphoenolpyruvate carboxykinase
PGC1α	Peroxisome proliferator-activated receptor gamma coactivator 1-alpha
TNFα	tumor necrosis factor-α
PPAR γ	Peroxisome proliferator-activated receptor gamma
AKT	Protein kinase B
OGTT	Oral glucose tolerance test
B.breve	Bifidobacterium breve
GAPDH	Glyceraldehyde-3-phosphatase dehydrogenase
AUC	Area under the curve
PCoA	principal coordinated analysis
LEfSe	linear discriminant analysis effect size
