# Supplementary material for: Lactate-Fortified Puerariae Radix Fermented by Bifidobacterium breve Improved Diet-Induced Metabolic Dysregulation via Alteration of Gut Microbial Communities
Source: Nutrients. 2020 Jan 21;12(2):276. doi: 10.3390/nu12020276 (PMC7070547; doi:10.3390/nu12020276)
Supplement: Supplementary file 1 [file nutrients-12-00276-s001.docx]

**Supplementary information**

**Lactate-fortified *Puerariae Radix* fermented by *Bifidobacterium breve* improved diet-induced metabolic dysregulation via alteration of gut microbial communities**

**Yura Choi^1^, Shambhunath Bose^2^, Narae Shin^1^, Young-Do Nam^3,4^, Eun-Ji Song^3,4^, Hojun Kim^1*^**

^1^Department of Rehabilitation Medicine of Korean Medicine, Dongguk University, 27 Donggukro, Ilsan-donggu, Goyang 10326, Republic of Korea

^2^BexPharm Healthcare Ltd., Seoul, Republic of Korea

^3^Research Group of Gut Microbiome, Korea Food Research Institute, Wanju-gun 24 Republic of Korea

^4^Department of Food Biotechnology, Korea University of Science and Technology, Wanju, Republic of Korea

*Corresponding author

Department of Rehabilitation Medicine of Korean Medicine, Dongguk University, 27 Dongguk-ro, Ilsan-donggu, Goyang 10326, Republic of Korea.

E-mail address: [kimklar@gmail.com](mailto:kimklar@gmail.com); [kimklar@dongguk.ac.kr](mailto:kimklar@dongguk.ac.kr)

Tel: +82 31 961 9111 Fax: + 82 31 961 9009


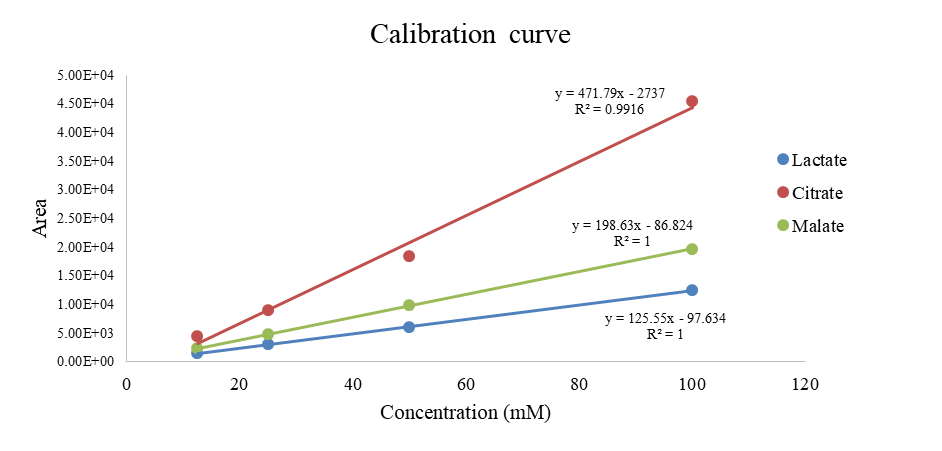


Figure S1. HPLC calibration curve of the organic acid standards.


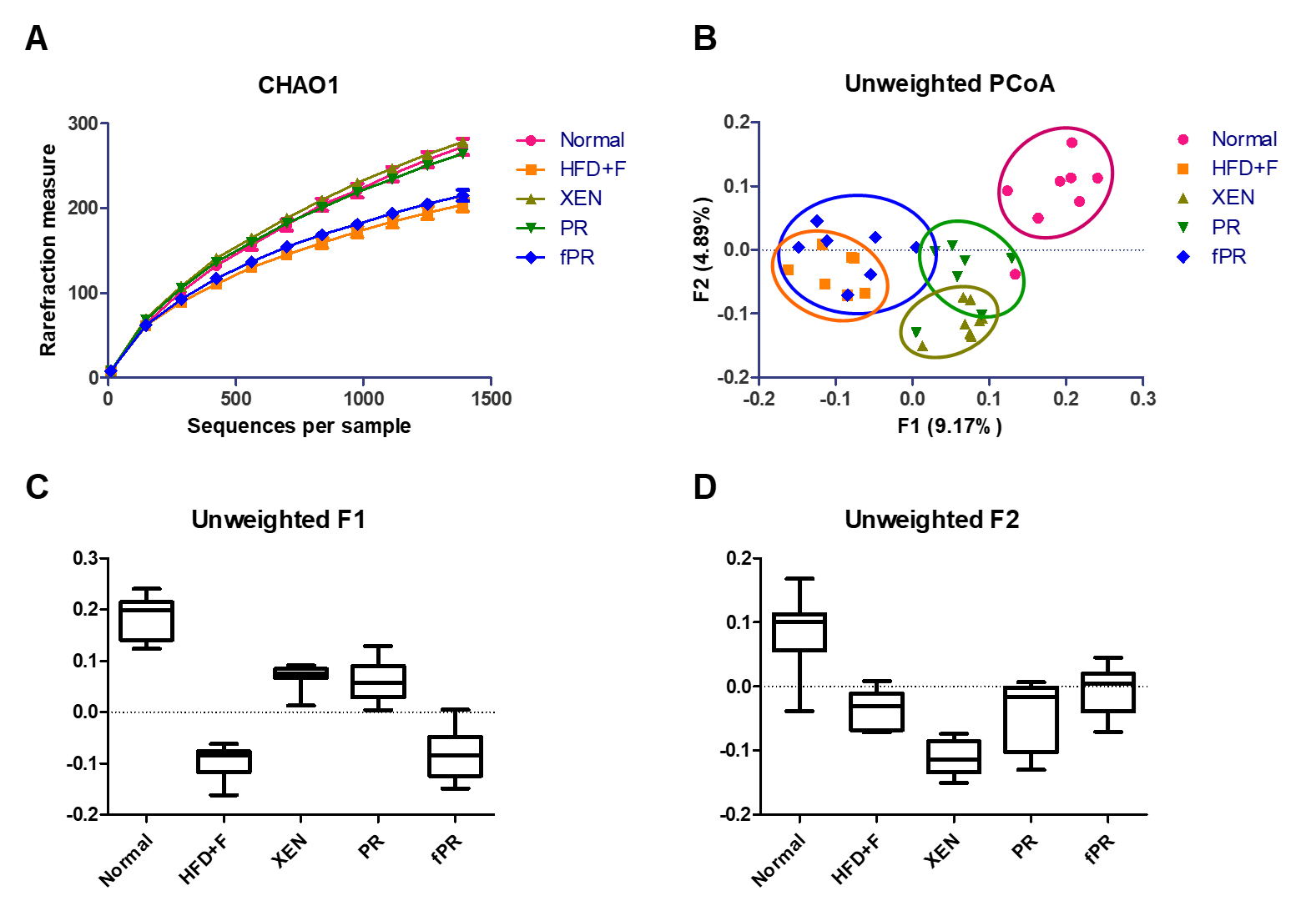


Figure S2. (A) Alpha-diversity, measured by determining the CHAO1 index. (B, C, D) PCoA score plots calculated from OTU levels by QIIME pipeline and subjected to unweighted UniFrac analysis.


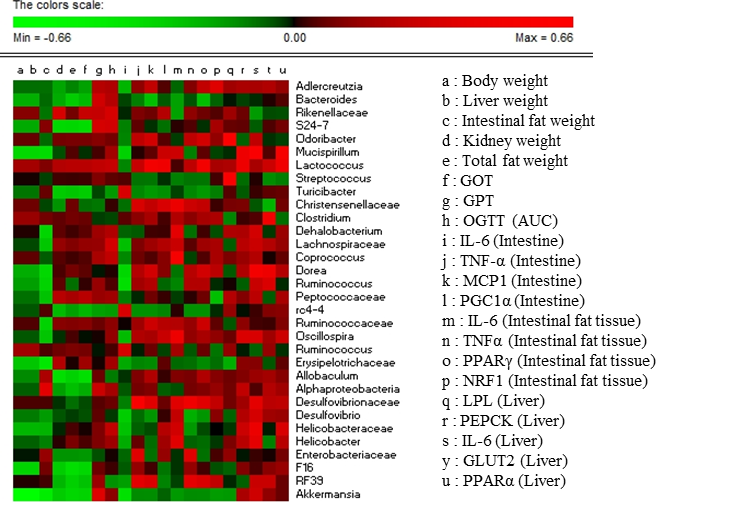


Figure S3. Hierarchical clustering presented as a heat-map shows the abundance of representative OTUs related to biomarkers (greatest difference between the gut microbiota and the biomarkers) selected for *p* < 0.05.

**Supplementary table**

| Gene | Forward | Reverse |
| --- | --- | --- |
| MCP1 | AAG AGA TCA GGG AGT TTG CT | CTG CCT CCA TCA ACC ACT TT |
| IL6 | CAT CCT CGA CGG CAT CTC AG | GCT CTG TTG CCT GGT CCT C |
| TNF α | GGT GAA GGG AAT GGG TAT | GGT CAC TGT CCC AGC ATC TT |
| PEPCK | GTC TAT GAA GCC CTC AGC T | AAG AAG GGT CGC ATG GCA A |
| PPAR γ | GCC TGT CTG TCG GGA TGT | GGC TTC GTG GAT TCT CTT |
| PGC1 α | AGC CGT GAC CAC TGA CAA CGA G | GCT GCA TGG TTC TGA GTG CTA AG |
| LPL | TTG CCC TAA GGA CCC CTG AA | ACA GAG TCT GCT AAT CCA GGA AT |
| NRF1 | CCC CCG AGG ACA CTT CTT ATG ATG | GGC CGT TTC CGT TTC TTC CCT GTT |
| GAPDH | GAC ATC AAG AAG GTG GTG AAG CAG | ATA CCA GGA AAT GAG CTT GAC AAA |

Table S1. Nucleotide sequences of primers used in quantitative real-time PCR
